# Editorial: Biomechanical performance and relevant mechanism of physical medicine and rehabilitation for neuromusculoskeletal disorders

**DOI:** 10.3389/fphys.2023.1290172

**Published:** 2023-10-31

**Authors:** Qipeng Song, Lin Wang, Pui Wah Kong, Feng Wei, Dongmei Wang, Li Li

**Affiliations:** ^1^ Department of Sport and Health, Shandong Sport University, Jinan, China; ^2^ School of Exercise and Health, Shanghai University of Sports, Shanghai, China; ^3^ Physical Education and Sports Science Academic Group, National Institute of Education, Nanyang Technological University, Singapore, Singapore; ^4^ Orthopaedic Biomechanics Laboratories, Michigan State University, East Lansing, MI, United States; ^5^ Biomechanics Laboratory College of Human Movement Science, Beijing Sport University, Beijing, China; ^6^ Department of Health Sciences and Kinesiology, Georgia Southern University, Statesboro, GA, United States

**Keywords:** rehabilitation, motor control, postural control impairment, falls, injury, postural stability, lower extremity injury

## Introduction

Biomechanics plays a crucial role in evaluating the effectiveness of physical medicine and rehabilitation for neuromusculoskeletal disorders. Assessments can be utilized for conditions such as degenerative dysfunction (e.g., falls or knee osteoarthritis in older adults) and sports-related injuries (e.g., ankle sprains or anterior cruciate ligament (ACL) injuries). Movements and daily functional activities of those who suffer injuries can be contrasted with their pre-injury condition or uninjured individuals. Pioneering investigations have taken a step forward by leveraging biomechanical metrics to formulate strategies within physical medicine and rehabilitation and delve into the underlying efficacy mechanisms (e.g., Li et al., 2019). Nonetheless, such inquiries remain somewhat scarce—this Research Topic aimed to stimulate the dissemination of more pertinent endeavors in this area.

The editorial team orchestrated this Research Topic to compile the advancements that elucidate the progress in physical medicine and rehabilitation for neuromusculoskeletal disorders. This Research Topic, “*Biomechanical Performance and Relevant Mechanism of Physical Medicine and Rehabilitation for Neuromusculoskeletal Disorders*,” encompassed 31 studies, comprising 24 original and seven review articles.

## Mechanisms of sports-related injuries

In sports-related injuries, the primary objective of injury prevention lies in minimizing injury risks and understanding the underlying mechanisms, particularly at knee and ankle joints. Among individuals who underwent anterior cruciate ligament reconstruction (ACLR), diminished postural control capability is a significant obstacle in their return to competitive activities (Wang et al.). A noteworthy observation was the reduced dynamic postural control exhibited by these participants, coupled with noticeable deficiencies in proprioception. Cui and group compared the surface electromyographic features of lower limb muscles in people with ACL injuries at different stages: 6 months before the injury, 6 months and 1 year after ACLR (Cui et al.). Their study could help determine which muscles require more training and which exercises are best suited for interventions. Based on the reports of retrospective studies, Grodman et al. observed that levels of activities or maneuvers during non-contact conditions among ACL-injured patients significantly increased 6 months after their injuries. Their findings provided evidence that changing levels of certain activities or maneuvers may play a role in ACL injury risk. Meanwhile, Long et al. utilized an electromyography (EMG) method to evaluate the risk of knee osteoarthritis following unilateral ACLR (Long et al.). The results showed that the peak tibial compression force, knee flexion and ankle dorsiflexion angles, and the muscle force of the rectus femoris and vastus medialis on the healthy side were greater than that on the surgical side during jogging. This indicated that the body automatically exhibited compensatory mechanisms on the healthy side to reduce the risk of further injury on the surgical side.

The lower extremity joints collectively form a tightly linked kinetic chain; therefore, an injury to one joint might cause compensation in another joints. Xu et al. reported that participants with chronic ankle instability (CAI) altered proximal lower extremity joint motion strategies during lateral cutting, jump landing, and abrupt stopping, potentially elevating the risk of ACL injury (Xu et al.). Kong’s group explored the effect of mental fatigue on biomechanical characteristics of lower extremities in people with functional ankle instability (FAI) (Kong et al.). They observed that the ankle stiffness of people with FAI had no significant change during anticipated side-step cutting but presented less ankle stiffness on the injured side during unanticipated side-step cutting to increase ankle instability and the risk of re-injury. Under conditions of visual deprivation, Meng et al. observed that individuals with FAI presented a greater risk of instability in the affected limb compared to the other limb (Meng et al.). The authors recommended that further research to investigate the influence of varying visual conditions on stability and gait performance.

## Rehabilitation strategies for sports injuries

This Research Topic included innovative methods and interventions, e.g., kinesio taping, electronic interventions, and machine learning. Li et al. utilized Kinesio Taping (KT) intervention on muscle strength and postural control in collegiate basketball athletes with FAI (Li et al.). They observed that the plantar flexor and dorsiflexor moment increased by 20% and 34%, respectively. Chen et al. explored the effects of KT therapy on gait and EMG in people suffering from stroke, showing that the root mean square of tibialis anterior EMG signals in the hemiplegia limbs decreased during walking after KT treatment (Chen et al.). Que et al. utilized the KT and vibration treatment (VT) to alleviate delayed-onset muscle soreness among college students (Que). They reported that KT and VT could reduce pain and strength loss to varying degrees. VT was better than KT in improving pain, and the combined intervention worked better than single interventions. The EMG-based robots were superior to conventional therapies in improving upper extremity motor control, spasticity, and activity limitation. Huo and coworkers showed that the efficacy of the treatment was better in people with post-stroke who received EMG-based robotic techniques combined with electrical stimulation (Huo et al.).

Virtual reality training improved lower extremity muscle strength and postural control. Wang et al. reported that an 8-week virtual reality training improves the muscle strength of hip flexors and extensors, knee flexors and extensors, and ankle plantar flexors (Wang et al.). However, no significant improvement was observed in postural control ability in adolescents with intellectual disability. Meanwhile, Gao et al. built a support vector machine to recognize the gait characteristics of both lower extremities before and after fatigue to prevent running injury, monitor movements, and assess gaits (Gao et al.). Based on continuous wavelet transform, Bai et al. reported that the impact force characteristics in the walking support period differed between people with and without flatfoot in the time and frequency domains (Bai et al.). Huang et al. showed that the sample entropy in the anteroposterior and mediolateral directions of the corrected vision state was greater than in myopia and eyes-closed conditions (Huang et al.). The maximum flexion angles of ankle and knee joints were in the following order: corrected vision, myopia, and eye-closed. That is, the stability of static and dynamic posture in corrected vision was worse than other vision conditions, and the activation and work of lower extremity muscles were increased. Meanwhile, Zeng et al. recruited 22 male college students with normal visual acuity to test gait characteristics at normal and with 150 ° and 450 ° concave lenses (Zeng et al.). They observed that hyperopic interventions impacted the kinematic gait characteristics in male college students, mainly in terms of altered postural control, increased instability, and increased difficulty in maintaining trunk stability with the risk of injury.

## Mechanisms and rehabilitation approaches of fall prevention

Fall prevention in older adults is a critically important Research Topic in the neuromusculoskeletal area, as falls can cause further health issues and lead to functional impairments (Peng et al., 2019). In this area, Wang and coworkers recruited 166 older adults in three age groups (young: 60–69 years, old: 70–79 years, and older: ≥ 80 years) and compared their differences in tactile sensation, proprioception, muscle strength, and postural control (Wang et al.). It was observed that the young and old adults had better postural control, tactile sensation, proprioception, and muscle strength compared to the older adults. Proprioception correlated with postural control in young and old adults but not in older adults. The worsened proprioception among the older could be the key to increased fall risks. Aging involves a decline in muscle and bone mass and a deterioration in cognitive function, especially during dual-task conditions (Song et al., 2018). Li et al. recruited 15 older adults to evaluate the test-retest reliability of kinematics and kinetics during single-task and dual-task stair walking (Li et al.). They observed that the intraclass correlation coefficient of kinematics and kinetics ranged from fair to excellent. These results may help researchers to access biomechanics of dual-task stair walking in the elderly and to interpret the effect of interventions in this population.

Physical activity plays a crucial role in the wellbeing of elderly individuals. Exercise intervention is an important approach to preventing falls, improving the quality of life, and alleviating pain symptoms in older adults (Song et al., 2018). Zhao et al. adopted typical Tai Chi to improve lower extremity inter-joint coordination and variability in older female adults (Zhao et al.). They observed that the mean absolute relative phases of hip-knee and knee-ankle and the deviation phase of hip-knee were significantly less in older adults who practiced Tai Chi than those who did not. Wang et al. reported the results of an 8-week Chinese herb hot compress combined with therapeutic exercise regarding people with knee osteoarthritis (Wang et al.). The intervention significantly relieved pain and decreased proprioception thresholds of knee extension and ankle plantarflexion. The results also showed improved functional performance, including the Time Up-and-Go test and 20-m walk test, compared to the therapeutic exercise training. Sun et al. described the role and regulation mechanism of Chinese traditional exercises, including Tai Chi, Baduanjin, Wuqinxi, and Yijin Jin, which had different emphases and could improve bone density in various parts of the body (Sun et al.). These exercises were found effective for older adults in improving the bone density of the exercisers and relieving pain, improving postural control, and regulating their psychological state. Moreover, neurological disorders with dyskinesia seriously affect older adults’ daily activities and are associated with the degeneration or injury of the musculoskeletal or nervous system (Song et al., 2021). Wu et al. described that most of the reviewed studies reported poor motor performance and higher cortical activation of Parkinson’s disease, stroke, and multiple sclerosis in older adults than healthy individuals (Wu et al.). More than 5 weeks of walking training or physiotherapy could promote motor function and cortical activation in people with Parkinson’s disease and stroke. Traditional Chinese exercises have been utilized to enhance adults’ physical and mental wellbeing (Song et al., 2018). Zhang et al. explored the effect of an 8-week Bafa Wubu of Tai Chi on college students. Anxiety and depression were significantly decreased in the Tai Chi group (Zhang et al.). The authors suggested these benefits could stem from modulating activity in the left middle frontal gyrus and the right middle frontal gyrus of the orbital part, respectively.

## Neuromusculoskeletal applications in sports performance

In other aspects of research on the neuromusculoskeletal area, injury prevention has consistently been a focal point in sports biomechanics. A front-view video analysis approach is a relatively safe, cost-effective tool for practitioners to measure foot inversion angles at the initial foot contact. Iskandar et al. further indicated that front-view foot inversion angles at the initial foot contact could be used to determine rearfoot inversion angles when crossover gait obstructs the back camera view (Iskandar et al.). A narrative review by Mei et al. suggested tracking foot motion across segments and converting 2D motion to 3D shape, using Machine Learning to address foot mechanics’ nonlinear links to shape or posture (Mei et al.). They suggested standardizing wearable data to quickly predict instant mechanics, load, injury risks, and foot tissue-bone adaptation. They further suggested correlating with shapes and analyzing real-life dynamic shapes and posture through markerless real-time methods for accurately evaluating clinical foot conditions and footwear development. A systematic review by Liu et al. reported the relationship between running economy and lower extremity stiffness, namely, vertical stiffness, leg stiffness, and joint stiffness (Liu et al.). They showed that endurance runners’ vertical, leg, and knee stiffness were negatively correlated with running economy. They suggested that maximum oxygen uptake and speed could be used to determine whether the runner could take full advantage of leg stiffness to minimize energy expenditure. Li et al. studied the effects of blood flow restriction training and electrical muscle stimulation during low-intensity squat training (Li et al.). The combined interventions improved the muscle strength of the lower extremities by promoting muscle hypertrophy and improving muscle activation. The authors suggested the results were likely due to such a combination compensated for the limitations and deficiencies of the two intervention methods when applied alone.

Moreover, Keriven et al. explored the effect of peripheral electromagnetic stimulation after an eccentric exercise-induced delayed-onset muscle soreness protocol in professional soccer players (Keriven et al.). They observed that stimulation did not significantly improve the lower extremities’ power and strength but decreased the vastus medialis’ peripheral sensitization after the eccentric exercise protocol. Female college students’ body postural control ability improved substantially after comprehensive sports activities (Zhang et al.). The participants had a more rational body composition and continued natural bone mineral density increase, unrelated to exercise intervention. Regarding athletic performance, Liang et al. reported that the lower extremity joint range of motion and stiffness significantly impacted the foot-up serve performance in tennis players (Liang et al.). A greater joint mobility and lower-limb stiffness promoted better performance during the foot-up serve preparation stage. For methodological considerations, Montoro-Bombú et al. provided information on criteria for determining the volume and intensity of drop jumps during plyometric training programs to help researchers conduct new plyometric training programs (Montoro-Bombú et al.).

This Research Topic aimed to encourage researchers to adopt biomechanics approaches to design advanced physical medicine and rehabilitation approaches. Researchers should also consider using biomechanical approaches to evaluate the effectiveness of the interventions and to explore the mechanisms by which rehabilitation programs work for neuromusculoskeletal disorders. This Research Topic expanded the application of biomechanics, promoted the development of research on neuromusculoskeletal systems, to better understanding and treating neuromusculoskeletal disorders.
